# Supplementary material for: In vivo imaging of phosphocreatine with artificial neural networks
Source: Nat Commun. 2020 Feb 26;11:1072. doi: 10.1038/s41467-020-14874-0 (PMC7044432; doi:10.1038/s41467-020-14874-0)
Supplement: Supplementary file 1 — Supplementary Information [file 41467_2020_14874_MOESM1_ESM.pdf]

Supplementary Information

In vivo imaging of phosphocreatine with artificial neural networks

Lin Chen[1,2], Michael Schär[2], Kannie W. Y. Chan[1,2,3], Jianpan Huang[3], Zhiliang Wei[1,2], Hanzhang Lu[1,2], Qin Qin[1,2], Robert G. Weiss[2,4], Peter C. M. van Zijl[1,2], Jiadi Xu[1,2,*]

[1] F.M. Kirby Research Center for Functional Brain Imaging, Kennedy Krieger Research Institute, Baltimore, MD, USA
[2] Russell H. Morgan Department of Radiology and Radiological Science, The Johns Hopkins University School of Medicine, Baltimore, MD, USA
[3] Department of Biomedical Engineering, City University of Hong Kong, Hong Kong, China
[4] Division of Cardiology, Department of Medicine, Johns Hopkins University School of Medicine, Baltimore, MD, USA

Supplementary Section 1. PCr and Cr phantom experiments as a function of saturation power and temperature.

Previous studies indicate that both PCr and Cr show discernible CEST peaks at 1.95 ppm (Cr and PCr) and 2.5 ppm (PCr) for skeletal muscle at high magnetic field strength.[1, 2, 3] However, at 3 T only one CEST peak at 2.5 ppm can be observed for human muscle even with optimized saturation parameters. To illustrate this, PCr and Cr phantom experiments (each 50 mM) with different saturation powers and temperatures were performed (Supplementary Figure 1). The exchange rate of exchangeable protons is temperature-dependent. At room temperature (21°C) at 3T, the Cr guanidinium protons still show a discernible peak at 1.95 ppm (Supplementary Figure 1c). However, with increasing temperature, the exchange rate increases and the Cr guanidinium peak coalesces with the water peak and no CrCEST peak can be observed at physiological temperature (37°C), which is consistent with previous CrCEST studies at 3T.[4, 5] On the other hand, over the entire 21°C to 37°C range studied, the PCr CEST Z-spectrum shows peaks at 2.5 ppm and 1.95 ppm that are assigned to the guanidinium and amide protons of PCr, respectively. Both peak intensities increase with temperature but do not change lineshape, which indicates that both peaks were in the slow exchange regime at 37°C, i.e., their exchange rates are much smaller than the frequency offsets in rad/s with respect to the water peak. The observed intensities of the two PCr peaks increase as a function of saturation power (Supplementary Figure 1d,e), but around 1.0 μT the 1.95 ppm peak starts to disappear under the width of the direct water saturation (DS). I*n vivo*, the increase of saturation power also augments the magnetization transfer contrast (MTC) signal from semi-solid macromolecules, which will scale down the observed CEST signal even more.[2] Notice that the peak intensity at 1.95 ppm is much weaker than that at 2.5 ppm and therefore more vulnerable to the MTC scale down effect. Finally, saturation competition due to overlap with the faster exchanging guanidinium protons of Cr further reduces the PCr amide peak at 1.95 ppm *in vivo*.

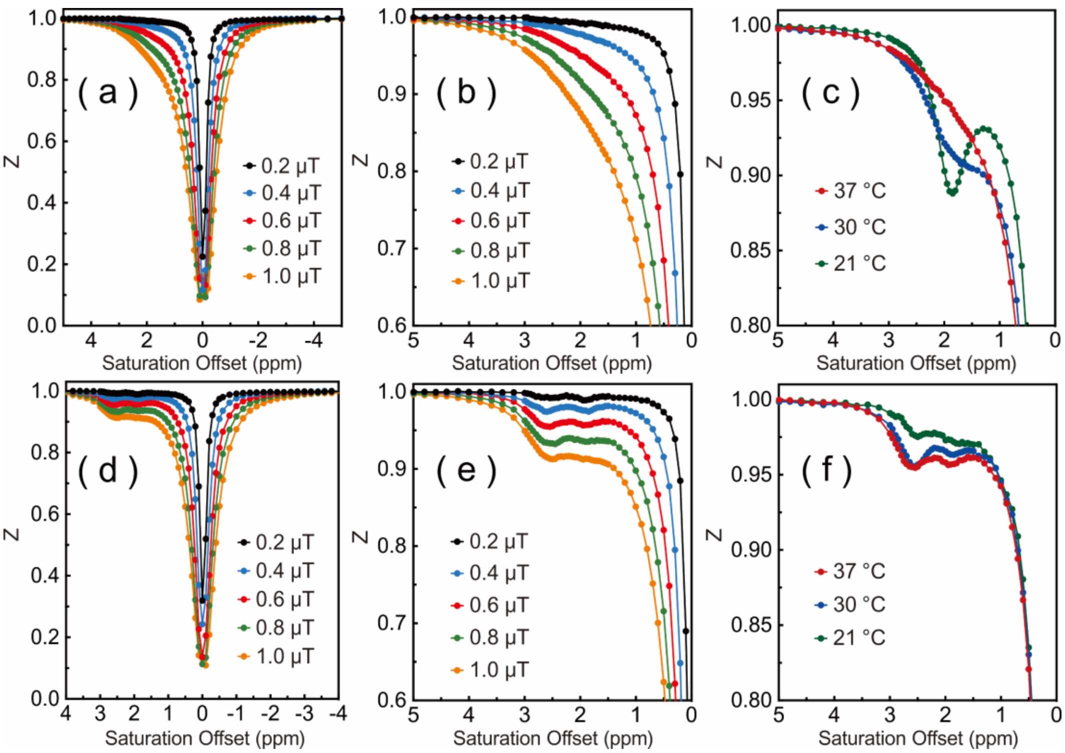

Supplementary Figure 1. Power and temperature dependence of the Z-spectra of 50mM Cr (a-c) and 50 mM PCr (d-f) at 3 T. The saturation power (B_1) dependence (a,b,d,e) was studied at 37˚C, with a saturation time of 1 s and B_1 ranging from 0.2 μT to 1 μT. Zoomed regions of the Cr and PCr Z-spectra in (a,d) are plotted in (b,e), respectively. CrCEST at 37˚C shows one broad shoulder around 1.95 ppm, while PCr CEST has two peaks, which are assigned to amide (1.95 ppm) and guanidinium (2.5 ppm) protons, respectively. (c) and (f) show Cr and PCr CEST Z-spectra, respectively, as a function of temperature (37 °C, 30 °C and 21 °C) for a saturation time of 800 ms and 0.6 μT saturation power.

Supplementary Section 2. PCr exchange rate measurement

The exchange rates of the two exchanging proton pools of PCr were measured on a Bruker 17.6T NMR spectrometer equipped with a triple resonance (TXI) NMR probe. A 20 mM PCr solution was prepared in phosphate buffered saline (PBS) and titrated to pH 7.3±0.1. Another set of phantoms was prepared by fixing the concentration at 20 mM for a pH dependence study (pH=6.7, 7, 7.3 and 7.6). The exchange rate was measured by the magnetization recovery of the exchangeable proton peaks after the inversion by a single selective Gaussian pulse (width of 2.5 ms). Then, the NMR signal was detected by a 3-9-19 WATERGATE sequence with a total echo time of 1 ms. Repetition time was 10 s. 15 inversion times from 0 ms to 60 ms were used for the

exchange rate measurement on the 1.95 ppm peak, while 15 inversion times from 0 ms to 20 ms were applied for the 2.6 ppm peak. The chemical shift of the guanidinium peak (2.6 ppm) at 17.6T is slightly higher than that at 3T (2.5 ppm), which may be due to some overlap with the amide proton peak at 3T. The recovery rate was determined by the apparent relaxation rate, $1/T_1 + k_{sw} \approx k_{sw}$, from the fitting of the recovery curve using the following equation

$$M = A - Be^{-k_{sw}TI} \qquad (SEq1)$$

where TI is the inversion recovery time. The typical inversion recovery curves for the two peaks at 37°C are plotted in Supplementary Figure 2, together with the curve fits from SEq1. The typical 17.6T NMR spectrum of PCr is also plotted in the insert, showing signals of the two exchanging proton pools, the residual water, and the aliphatic groups. The exchange rates for these PCr protons at pH = 7.3 are presented as a function of temperature in Supplementary Figure 2b. At 37°C, they were determined to be 260±40 Hz (2.6 ppm) and 122±10 Hz (1.95 ppm), consistent with the observed line width difference for these two peaks in the NMR spectrum, (150±10 Hz for 2.6 ppm vs 55±5 Hz for 1.95 ppm), i.e. the linewidth is broader with faster exchanging protons (see Supplementary Figure 2a). The pH dependencies of the exchange rates at 37°C are plotted in Supplementary Figure 2c. The exchange rate ratio between the two peaks for different temperatures (at pH = 7.3) and pH (at 37°C) are plotted in Supplementary Figure. 2d,e), respectively, and show a very narrow range between 1.5 and 2.1.

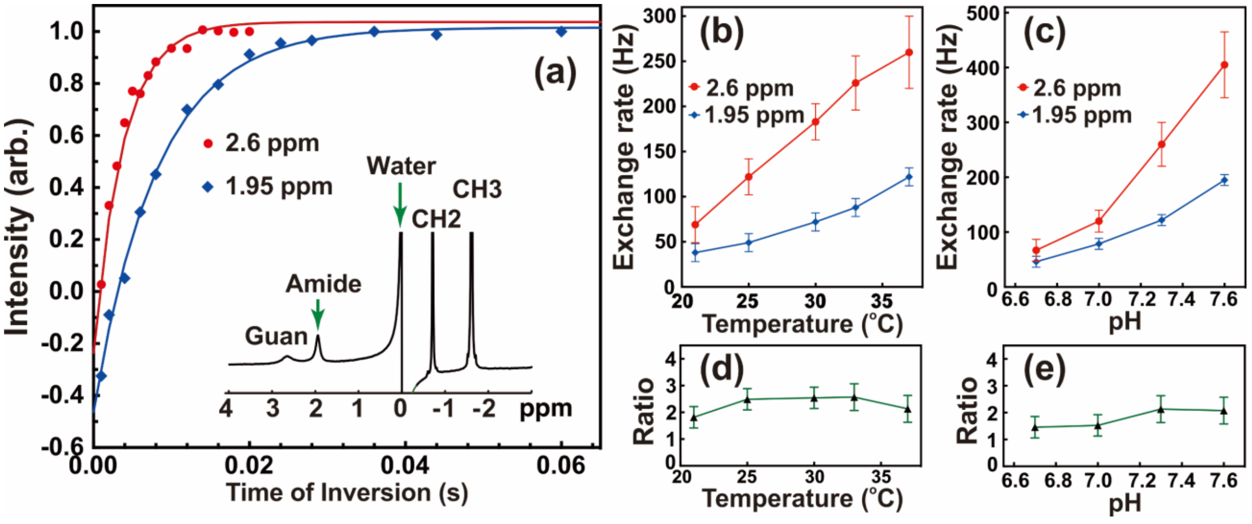

Supplementary Figure 2. (a) The inversion recovery curves of peaks at 1.95 ppm (blue) and 2.6 ppm (red). These curves were fitted using a single exponential curve (Eq. SEq1). The NMR spectrum of 20 mM PCr solution recorded at 17.6 T using a WATERGATE sequence is plotted in

the inset. Temperature (b) and pH (c) dependence of the exchange rates for the PCr CEST peaks at 2.6 ppm and 1.95 ppm, respectively. The exchange rate ratio between the peaks at 2.6 ppm and 1.95 ppm as functions of temperature and pH are plotted in (d) and (e), respectively. The temperature dependence experiments were performed with a pH value of 7.3, while the pH dependence experiments were performed at 37 ℃.

Supplementary Table 1: The concentration and exchange rate values (mean ± s.d.) obtained using ANNCEST and Bloch fitting for the simulated Z-spectra in Figure 1 in the main text. Ground truth is in the dark-colored rows.

Concentration(mM)	10	20	30	40	50
ANNCEST	10.3±0.3	19.9±0.3	30.1±0.4	40.0±0.3	49.9±0.3
Bloch Fitting	9.3±1.5	20.4±0.7	28.0±0.9	40.0±0.2	50.3±0.2
Exchange Rates at 2.5 ppm(Hz)	180	300	140	220	260
ANNCEST	180±12	301±6	141±5	220±3	260±3
Bloch Fitting	109±11	330±22	195±33	247±3	285±2

Supplementary Table 2: The exchange rates and concentrations (mean ± s.d.) obtained using ANNCEST on the PCr phantoms shown in Figure 2 in the main text. Concentration ground truth is in the darker-colored row.

Phantoms	10 mM	20 mM	30 mM	40 mM	50 mM
Concentration (mM)	9.2±1.2	20.9±1.1	30.3±1.0	39.2±1.2	50.5±1.6
Exchange Rate at 2.5 ppm (Hz)	239 ± 27	232 ± 14	243 ± 11	242 ± 7	255 ± 8

Supplementary Section 3. Parameters for generating training data and statistical evaluation of the performance of the neural network

Supplementary Table 3: Parameters used to generate training data for PCr phantom at 3T

**Saturation power**	0.6 µT
**Saturation length**	10 s
**Z-spectral range**	0.5 - 4 ppm
T_{1w}	2.6 s
T_{2w}	1.8 s
$T_{1(PCr\ protons)}$	0.05 s
$T_{2(PCr\ protons)}$	0.02 s
**CEST peaks**	1.95 ppm, 2.5 ppm
**Exchange rate ratio**	1.95 ppm : 2.5 ppm = 1 : 2.19
**Exchange rate range**	50 - 200 Hz
**Concentration ratio**	1.95 ppm : 2.5 ppm = 1 : 2
**Concentration range**	5 - 85 mM
**B_0 inhomogeneity range**	-0.4 - 0.4 ppm
**Noise**	Gaussian white noise with zero mean value and 0.0015 standard deviation

Supplementary Table 4: The parameters used to generate training data for human skeletal muscle at 3 T

**Saturation power**	0.6 μT
**Saturation length**	800 ms
**Z-spectral range**	1.3 - 3.5 ppm
T_{1w}	1.2 s
T_{2w}	15 - 35 ms
$T_{1(PCr\ protons)}$	0.05 s
$T_{2(PCr\ protons)}$	0.02 s
**CEST peak**	2.5 ppm
**Exchange rate range**	80 - 230 Hz
**Concentration range**	0 - 100 mM
**B_0 inhomogeneity range**	-0.25 - 0.25 ppm
**B_1 inhomogeneity range**	0.5 - 0.7 μT
**MTC/background concentration**	8 M
**MTC/background exchange rate**	30 Hz
**MTC/background T_1**	1 s
**MTC/background T_2**	9.1×10^{-6} s
**MTC/background lineshape**	Super Lorentzian
**Noise**	Gaussian white noise with zero mean value and 0.0035 standard deviation

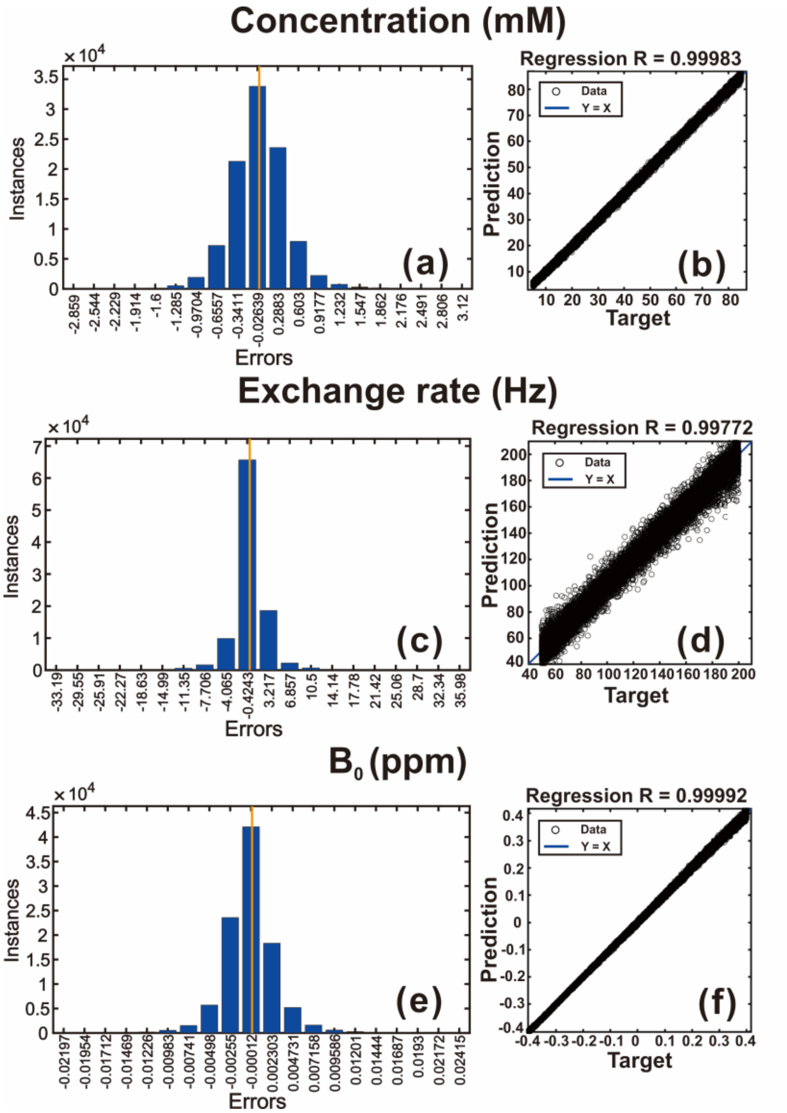

Supplementary Figure 3. Error histogram and regression plot of the neural network training results for numerical simulation and phantom experiment. For the error histogram, the horizontal axis is the absolute error between predicted outcomes and targeted values, and the vertical axis stands for the number of elements in different ranges. The linear regression *R* values were calculated between predicted outcomes and targeted values. The ideal *R*-value is 1, which means the predicted outcomes and targeted values are the same.

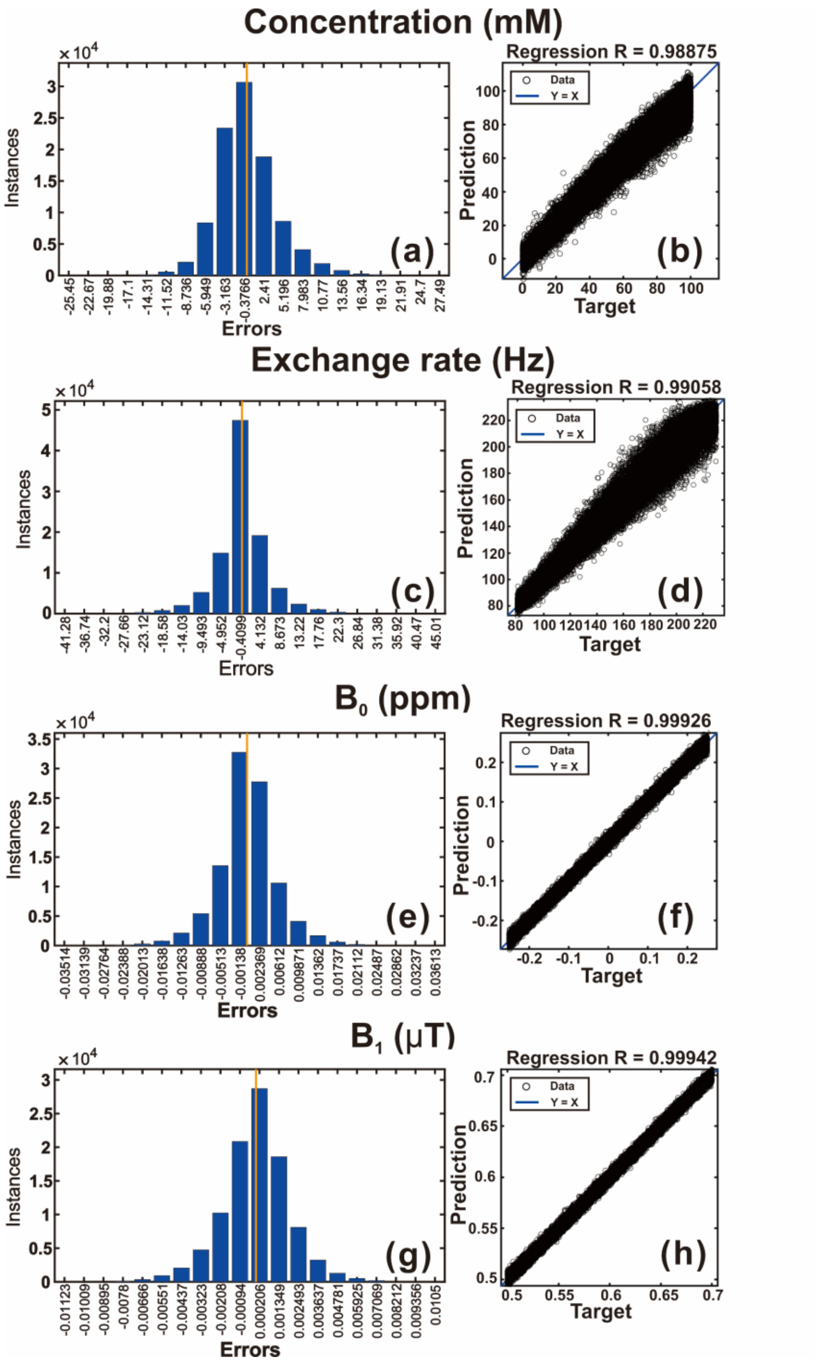

Supplementary Figure 4. Error histogram and regression plot of the neural network training results for human muscle experiments.

Supplementary Section 4. Simulated Z-spectra of human skeletal muscle with various concentrations, exchange rates, B_0 and B_1

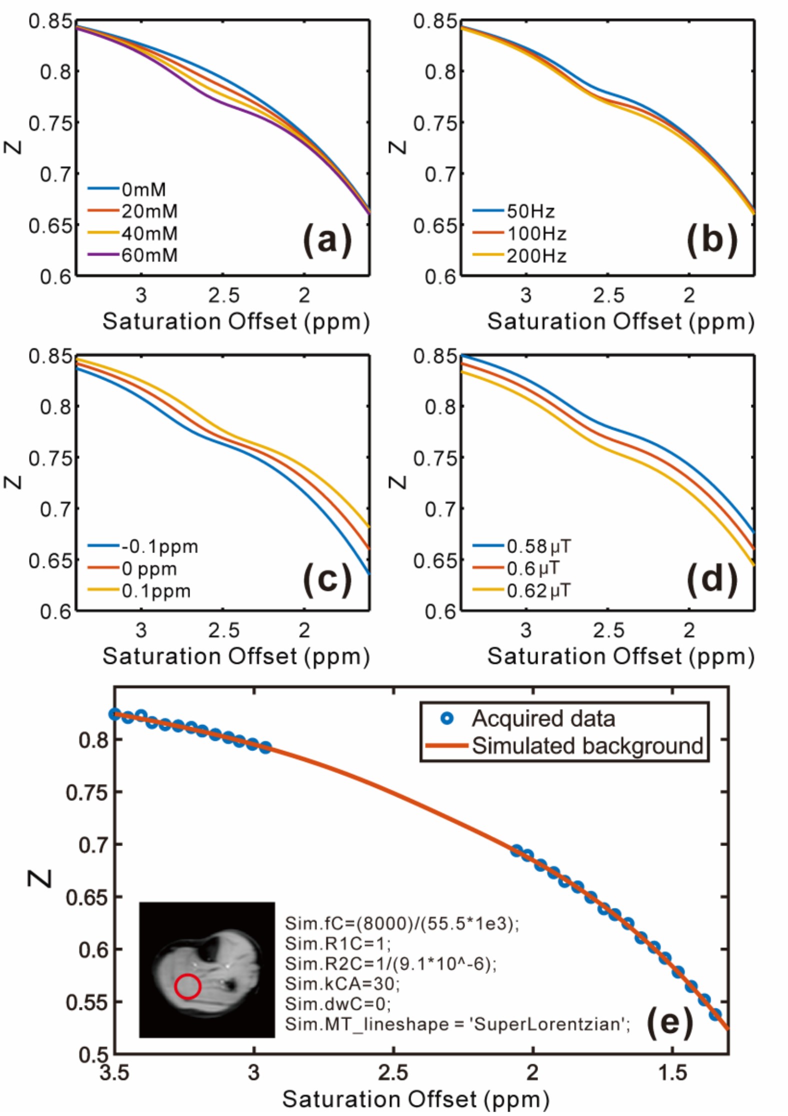

Supplementary Figures 5: The simulated in vivo CEST Z-spectra using three-pool Bloch-McConnell equations, i.e. water, MTC and PCr pool. The default concentration, exchange rate, B_1 and B_0 were 60 mM, 200 Hz, 0.6 μT and 0 ppm, respectively. Z-spectra with different (a) concentrations, (b) exchange rates, (c) B_0 values and (d) saturation powers. (e) The MTC dominant background can be well presented by a pool with a mean concentration of 8 M and mean exchange rate of 30 Hz.

Supplementary Section 5. Spatially resolved PCr recovery time map

As a proof-of-principle application, the PCr recovery rate map was calculated for one

typical subject and shown in Supplementary Figure 6. To compensate for the sparsity of sampling time points, the baseline data was inserted behind the last recovery data as an additional time point. The time interval of PCr mapping was 90 s. The PCr recovery time constant τ_{PCr} was fitted using the following equation[6]:

$$PCr(t) = PCr_0 + \Delta PCr \cdot (1 - e^{-t/\tau_{PCr}}) \qquad \text{(SEq2)}$$

where PCr_0 is the PCr concentration at the end of exercise, ΔPCr refers to the difference in PCr concentration between resting and end of exercise. The fitting was accomplished by the MATLAB built-in function "lsqcurvefit". The fitting ranges of PCr_0, ΔPCr and τ_{PCr} were set to 1~ 50 mM, 1~ 50 mM and 1 ~ 300 s, respectively.

From the fitting result shown in Supplementary Figure 6b, PCr concentrations of gastrocnemius muscles show exponential recovery after exercise and the fitted τ_{PCr} values 72.6 s is consistent with that obtained by ^{31}P MR (63.1 ± 25.9 s)[7]. However, except for the gastrocnemius muscles, the PCr recovery of other muscles could not be well captured and fitted due to the coarse temporal resolution (Supplementary Figure 6c). Improving the temporal resolution of PCr ANNCEST may yield a more detailed and robust τ_{PCr} map, which needs further study.

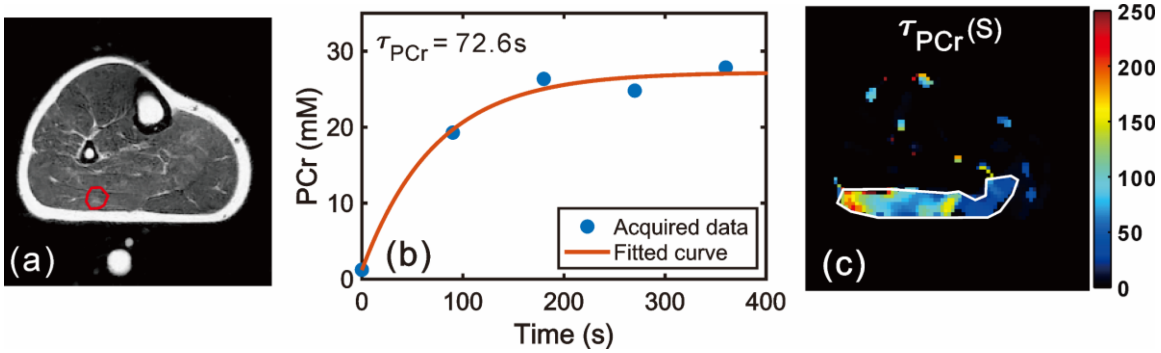

Supplementary Figure 6. Proof-of-principle calculation of the PCr recovery time constant τ_{PCr}. (a) T_2 weighted anatomical image and selected ROI (red circle). (b) The fitting results of the selected ROI indicated in (a). (c) Fitted τ_{PCr} map corresponding to PCr ANNCEST data shown in Figure 5. The fitted τ_{PCr} values within the regions circumscribed by the white line are 70.7 ± 55.4 s (mean ± s.d.).

Supplementary Section 6. Feasibility of ANNCEST in quantifying other metabolites

ANNCEST is a data-driven quantification method, which is designed to extract relevant features from Z-spectra and utilize them to create a predictive tool based on the pattern hidden inside. The flowchart of ANNCEST is shown in Supplementary Figure 7.

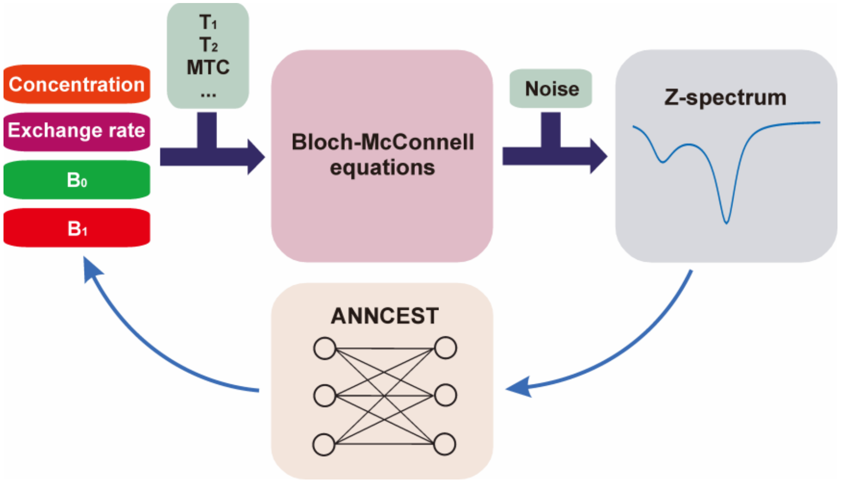

Supplementary Figure 7. Schematic flowchart of ANNCEST. The annotated Z-spectra are simulated by Bloch-McConnell equations with proper assumptions and parameters that mimic realistic tissue conditions. ANNCEST is trained to extract relevant features between Z-spectrum and quantifiable parameters. During neural network training, only Z-spectra and quantifiable parameters are provided, and the other parameters (e.g. T_1, T_2, MTC, and noise) are blind for the neural network.

In order to demonstrate that ANNCEST is valid for other metabolites with a different number of proton pools and saturation length, Cr phantom experiments were performed. The training data for these experiments were generated using the Bloch-McConnell equations. The frequency offsets of the Z-spectra ranged from 0.5 to 4 ppm with a total offset number of 50. The offsets of Cr CEST peaks were set to 1.95 ppm. The T_1 and T_2 of water protons were set to 2.6 s and 1.8 s, respectively, according to the measurements on the phantom. The saturation power and duration were 0.6 μT and 3 s. The concentration, exchange rate, and B_0 inhomogeneity were chosen randomly from the ranges of 5 to 105 mM, 100 to 350 Hz, -0.2 to 0.2 ppm, respectively. Gaussian white noise with zero mean value and 0.0015 standard deviation was imposed on the simulated Z-spectra. The number of Z-spectra used for neural network training was 105. The training results are shown in Supplementary Figure 8, which reflects that ANNCEST can find strong correlations between Z-spectra and quantifiable parameters. For validation, we applied the trained ANNCEST to quantify Z-spectra of Cr phantom obtained at room temperature (25℃), and the results are given in Supplementary Figure 9. An excellent correlation (R = 0.9996) was observed between the ground truth and predicted phantom Cr concentration. The related Bland-Altman analysis of concentration is shown in Supplementary Figure 9f. The exchange rate obtained by ANNCEST (237.8 ± 17.6 Hz) was consistent with that from the previous study[8] (239~301 Hz at 25℃, pH 6.9-7.0). The predicted B_0 map (Supplementary Figure 9e) showed a strong correlation (0.9851) with that obtained by water saturation shift referencing (WASSR) MRI, as illustrated in Supplementary Figure 9h.

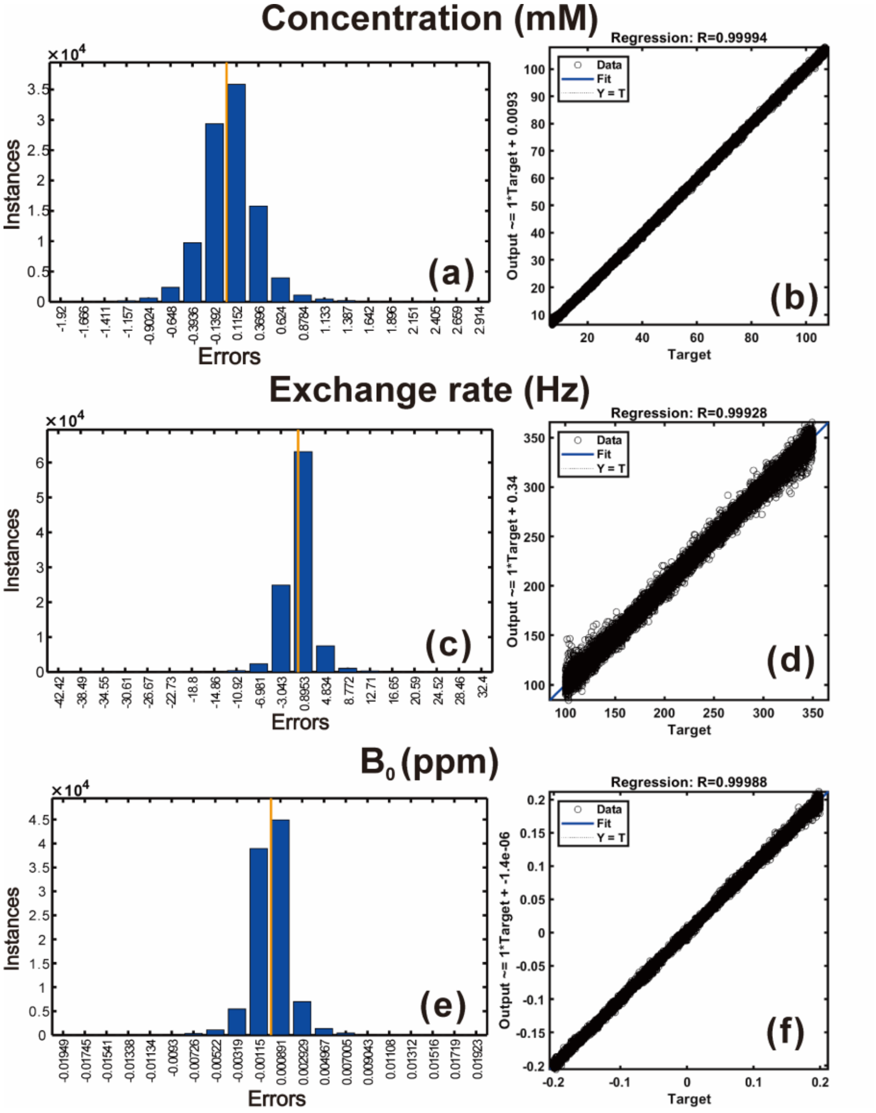

Supplementary Figure 8. Error histogram and regression plot of the neural network training results for Cr phantom experiment.

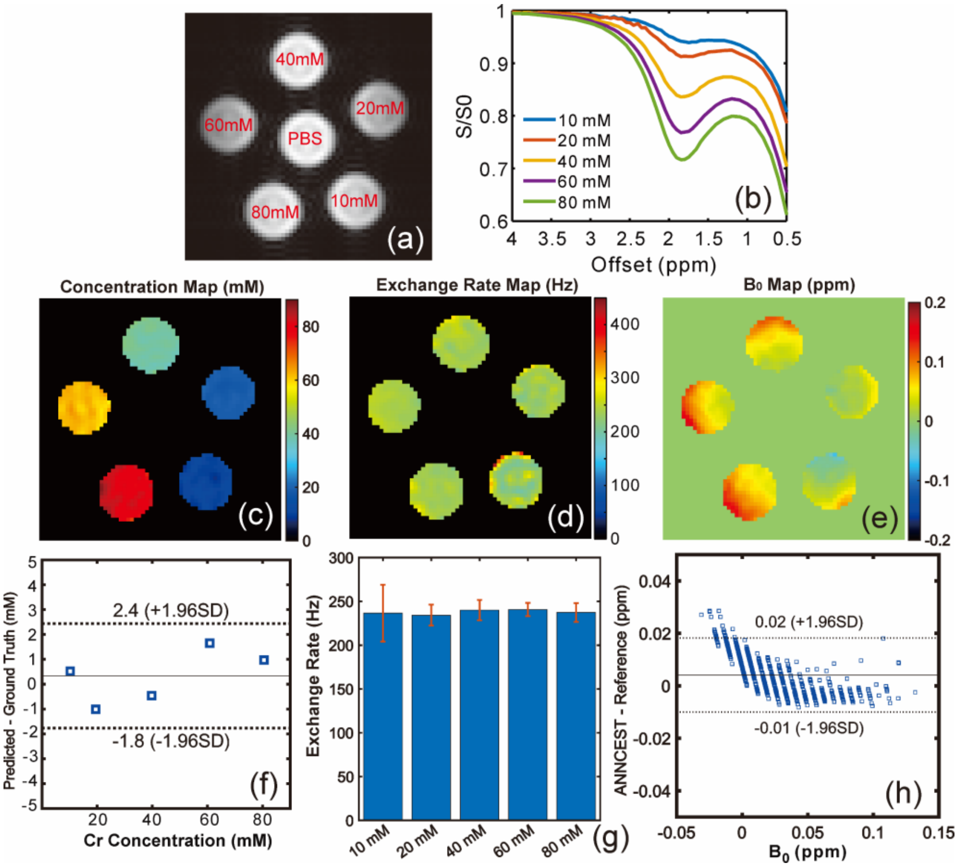

Supplementary Figure 9. Typical Validation of ANNCEST at 3T (preclinical MRI) on a phantom consisting of test tubes with different concentrations of Cr. All experiments were performed at room temperature (25 ℃). (a) The arrangement of the Cr phantoms with different concentrations. (b) Representative Z-spectra extracted from one pixel of each of the Cr phantoms. The ANNCEST-predicted concentration (c), exchange rate at 1.95 ppm (d) and B_0 maps (e) for the CEST experiments collected using a 3 s saturation pulse of 0.6 μT. (f) Bland-Altman plot for the predicted concentration and ground truth. (g) The exchange rate quantification results. The bar and error bar indicate the mean value and standard deviation across each phantom, respectively. (h) Bland-Altman plot for the predicted B_0 map and referenced B_0 map obtained via WASSR method.

Supplementary Section 7. Power analysis for ANNCEST and ^{31}P 2D MRS

In this study, PCr mapping using ANNCEST was validated by comparison with ^{31}P 2D MRSI measures obtained before and during in-magnet plantar flexion exercise. A power analysis was performed to determine the appropriate data size to draw the conclusion. Assume we accept a $p < 0.001$ as acceptable and a study with 95% power, the sample size for the study will be[9]:

$$n = \frac{2\times(3.2905+1.6449)^2\sigma^2}{\Delta^2} \qquad \text{(SEq3)}$$

where σ refers to the estimated standard deviation and Δ indicates the difference in effect. In this study, we expected a 50% reduction in PCr concentration during exercise (i.e. $\Delta \approx 15$ mM), and the standard deviation of PCr concentrations is 7.84 mM based on the baseline data shown in Figure 5 (c). According to Eq. SEq3, the required sample size is about 14. In this study, a pixel-by-pixel correlation analysis was performed to compare PCr maps obtained by ANNCEST and ^{31}P 2D MRS on resting and exercised human skeletal muscle. Each PCr map has 16×16 = 256 pixels, and the PCr maps of baseline, during holding, and 0.75 min of recovery were chosen. Even though only partial regions were chosen for correlation analysis, the effective data size from four subjects is 202, which is much larger than the required sample size.

Supplementary Section 8. Validation of ANNCEST with different T_1 and T_2 values

In order to validate the robustness of ANNCEST against water T_1 and T_2 variations, we applied well-trained ANNCEST to quantify simulated Z-spectra with different water T_1 and T_2 values. From Supplementary Figure 10b, the concentration obtained by ANNCEST exhibits a slightly linear correlation with water T_1 values. When increasing water T_1 from 1.0 s to 2.0 s, the quantified concentration increased from 35.03 ± 1.95 mM to 37.33 ± 1.90 mM. The exchange rate obtained by ANNCEST possesses excellent resistance against water T_1 variation (160.7± 2.6 Hz at T_1=1 s v.s. 159.0±3.3 Hz at T_1 =2 s), as shown in Supplementary Figure 10c. The quantified concentration and exchange rate with different water T_2 values are shown in Supplementary Figure 10e,f. From the results, ANNCEST still can yield satisfactory accuracy when water T_2 varies from 15 ms to 50 ms, e.g. concentration varies between 34.6 mM and 36.5 mM, while the exchange rate is in the range of 160.1 Hz to 168.2 Hz. The ranges used here are sufficient to cover water T_1 and T_2 changes during exercise[10].

The robustness of ANNCEST against T_1 and T_2 variations of PCr proton is shown in Supplementary Figure 11. From the results, the concentrations and exchange rates obtained by ANNCEST possess excellent resistance against T_1 variation of PCr proton (34.75 ± 2.35 mM and 163.7 ± 3.5 Hz at 30 ms v.s. 36.06 ± 2.16 mM and 158.6 ± 4.9 Hz at 70 ms). Similarly, ANNCEST still can yield satisfactory accuracy when the PCr proton T_2 varies from 15 ms to 25 ms, giving a concentration variation between 34.9 mM and 36.9 mM, while the exchange rate ranges from 162.8 Hz to 159.0 Hz.

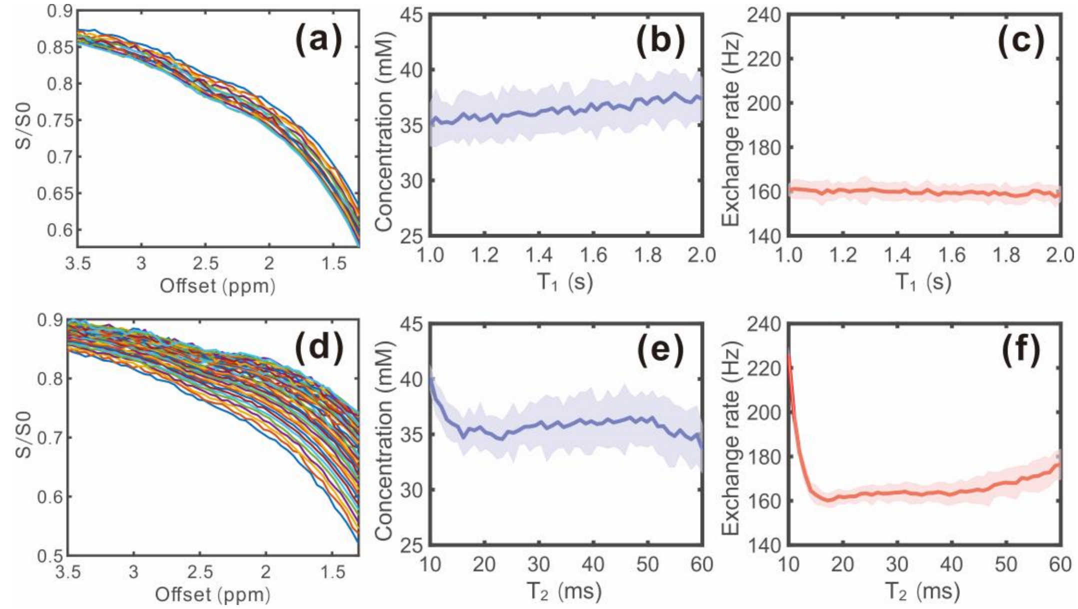

Supplementary Figure 10. Simulations showing that ANNCEST for PCr gives reasonable results over the relevant *in vivo* range of water T_1 and T_2 values. The gold standard concentration, exchange rate, B_0, and B_1 were set to 35 mM, 160 Hz, 0 ppm and 0.6 μT. The other parameters are listed in Supplementary Table 4. The simulations were repeated 50 times with the amount of noise added being varied. (a) Representative Z-spectra with water T_1 values ranging from 1.0 to 2.0s. The concentrations (b) and exchange rates (c) obtained by ANNCEST as a function of water T_1. (d) Representative Z-spectra for water T_2 values ranging from 10 to 60 ms. The concentrations (e) and exchange rates (f) obtained by ANNCEST as a function of water T_2. The solid line refers to the mean value of 50 repetitions and the light-colored area represents the standard deviation.

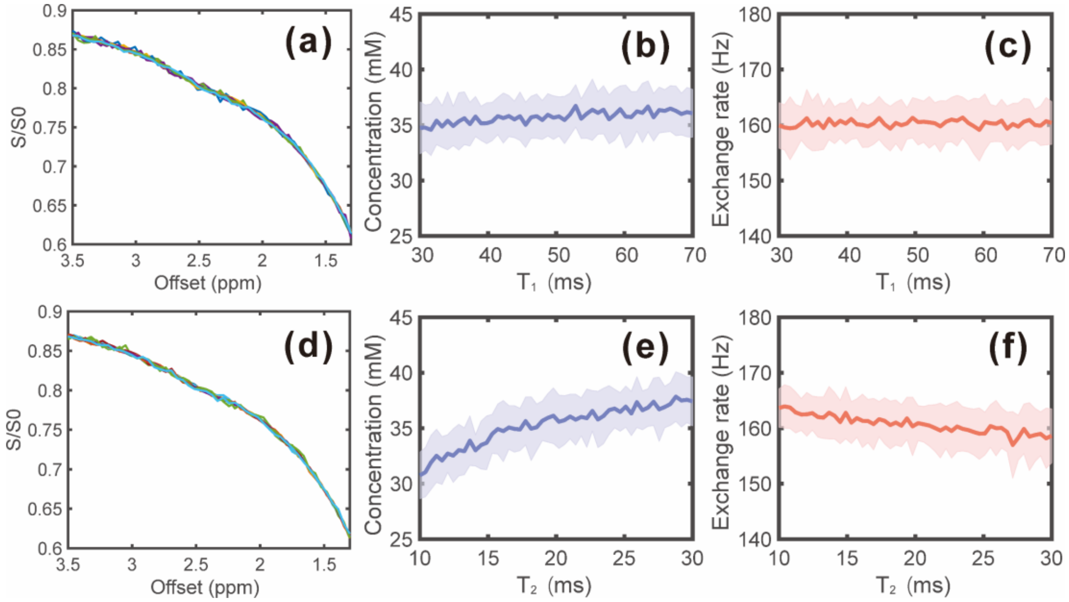

Supplementary Figure 11. Simulations showing that ANNCEST for PCr gives reasonable results over a range of T_1 and T_2 values for PCr protons. The gold standard concentration, exchange rate, B_0, and B_1 were set to 35 mM, 160 Hz, 0 ppm and 0.6 μT. The other parameters are listed in Supplementary Table 4. The simulations were repeated 50 times with the amount of noise added being varied. (a) Representative Z-spectra with water T_1 values ranging from 30 to 70 ms. The concentrations (b) and exchange rates (c) obtained by ANNCEST as a function of T_1 of the PCr protons. (d) Representative Z-spectra with PCr proton T_2 values ranging from 10 to 30 ms. The concentrations (e) and exchange rates (f) obtained by ANNCEST as a function of PCr proton T_2. The solid line refers to the mean value of 50 repetitions and the light-colored area represents the standard deviation.

References

1. Chen L, Barker PB, Weiss RG, van Zijl PCM, Xu J. Creatine and phosphocreatine mapping of mouse skeletal muscle by a polynomial and Lorentzian line-shape fitting CEST method. Magn Reson Med 81, 69-78 (2019).

2. Chen L, et al. Investigation of the contribution of total creatine to the CEST Z-spectrum of brain using a knockout mouse model. NMR Biomed 30, e3834 (2017).

3. Chung JJ, Jin T, Lee JH, Kim SG. Chemical exchange saturation transfer imaging of phosphocreatine in the muscle. Magn Reson Med 81, 3476-3487 (2019).

4. Haris M, et al. Exchange rates of creatine kinase metabolites: feasibility of imaging creatine by chemical exchange saturation transfer MRI. NMR Biomed 25, 1305-1309 (2012).

5. Haris M, et al. A technique for in vivo mapping of myocardial creatine kinase metabolism. Nat Med 20, 209-214 (2014).

6. Isbell DC, et al. Delayed calf muscle phosphocreatine recovery after exercise identifies peripheral

arterial disease. J Am Coll Cardiol 47, 2289-2295 (2006).

7. Schmid AI, et al. Dynamic PCr and pH imaging of human calf muscles during exercise and recovery using (31) P gradient-Echo MRI at 7 Tesla. Magn Reson Med 75, 2324-2331 (2016).

8. Goerke S, Zaiss M, Bachert P. Characterization of creatine guanidinium proton exchange by water-exchange (WEX) spectroscopy for absolute-pH CEST imaging in vitro. NMR Biomed 27, 507-518 (2014).

9. Kadam P, Bhalerao S. Sample size calculation. Int J Ayurveda Res 1, 55-57 (2010).

10. Marty B, Carlier PG. Physiological and pathological skeletal muscle T1 changes quantified using a fast inversion-recovery radial NMR imaging sequence. Sci Rep 9, 6852 (2019).
