# Supplementary material for: In vivo imaging of phosphocreatine with artificial neural networks
Source: Nat Commun. 2020 Feb 26;11:1072. doi: 10.1038/s41467-020-14874-0 (PMC7044432; doi:10.1038/s41467-020-14874-0)
Supplement: Supplementary file 3 — Description of Additional Supplementary Files [file 41467_2020_14874_MOESM3_ESM.docx]

Description of Additional Supplementary Files

File Name: Supplementary Data 1

Description: This file contains the code used in this study.
