# Supplementary material for: In vivo imaging of phosphocreatine with artificial neural networks
Source: Nat Commun. 2020 Feb 26;11:1072. doi: 10.1038/s41467-020-14874-0 (PMC7044432; doi:10.1038/s41467-020-14874-0)
Supplement: Supplementary file 5 — Reporting Summary [file 41467_2020_14874_MOESM5_ESM.pdf]

nature research

Corresponding author(s): NCOMMS-19-24815

Last updated by author(s): Aug 21, 2019

Reporting Summary

Nature Research wishes to improve the reproducibility of the work that we publish. This form provides structure for consistency and transparency in reporting. For further information on Nature Research policies, see Authors & Referees and the Editorial Policy Checklist .

Statistics

For all statistical analyses, confirm that the following items are present in the figure legend, table legend, main text, or Methods section.

n/a	Confirmed	
☐	☒	The exact sample size (*n*) for each experimental group/condition, given as a discrete number and unit of measurement
☐	☒	A statement on whether measurements were taken from distinct samples or whether the same sample was measured repeatedly
☐	☒	The statistical test(s) used AND whether they are one- or two-sided<br>*Only common tests should be described solely by name; describe more complex techniques in the Methods section.*
☐	☒	A description of all covariates tested
☐	☒	A description of any assumptions or corrections, such as tests of normality and adjustment for multiple comparisons
☐	☒	A full description of the statistical parameters including central tendency (e.g. means) or other basic estimates (e.g. regression coefficient) AND variation (e.g. standard deviation) or associated estimates of uncertainty (e.g. confidence intervals)
☐	☒	For null hypothesis testing, the test statistic (e.g. *F*, *t*, *r*) with confidence intervals, effect sizes, degrees of freedom and *P* value noted<br>*Give P values as exact values whenever suitable.*
☒	☐	For Bayesian analysis, information on the choice of priors and Markov chain Monte Carlo settings
☒	☐	For hierarchical and complex designs, identification of the appropriate level for tests and full reporting of outcomes
☒	☐	Estimates of effect sizes (e.g. Cohen's *d*, Pearson's *r*), indicating how they were calculated

Our web collection on statistics for biologists contains articles on many of the points above.

Software and code

Policy information about availability of computer code

Data collection	The images on phantoms were collected using Bruker Topspin 3.0 and Paravisison 6.0 software, while the human study were collected using Phillips Achieva 5.1 and Ingenia 5.4 systems.
Data analysis	The images were processed using custom code on MATLAB platform (www.mathworks.com, version 9.4.0.813654). The code for generating the images was deposited to GitHub (https://github.com/LinChenMRI/ANNCEST).

For manuscripts utilizing custom algorithms or software that are central to the research but not yet described in published literature, software must be made available to editors/reviewers. We strongly encourage code deposition in a community repository (e.g. GitHub). See the Nature Research guidelines for submitting code & software for further information.

Data

Policy information about availability of data

All manuscripts must include a data availability statement. This statement should provide the following information, where applicable:

- Accession codes, unique identifiers, or web links for publicly available datasets
- A list of figures that have associated raw data
- A description of any restrictions on data availability

The source data underlying Figs. 1d-n, 2c-g, 3a-i, 4b-h, 5b-g are provided as a Source Data file. The other data that support the findings of this study are available from the corresponding author upon reasonable request.

Field-specific reporting

Please select the one below that is the best fit for your research. If you are not sure, read the appropriate sections before making your selection.

Life sciences study design

All studies must disclose on these points even when the disclosure is negative.

Sample size	Power analysis was performed to determine the appropriate data size to draw the conclusion. Assume we accept a $p < 0.001$ as acceptable and a study with 95% power, the sample size for the study will be about 14. In this study, a pixel-by-pixel correlation analysis was performed to compare PCr maps obtained by ANNCEST and 31P 2D MRS on resting and exercised human skeletal muscle. Each PCr map has 16×16 = 256 pixels, and the PCr maps of baseline, during holding, and 0.75 min of recovery were chosen. Though only partial regions were chosen for correlation analysis, the effective data size from four subjects is 202, which is much larger than the required sample size.
Data exclusions	No subjects were excluded for the analysis.
Replication	All subject were measured successfully.
Randomization	This is not relevant to our study since not group comparison were perform.
Blinding	The phosphocreatine maps collected 31P MRSI and CEST methods were blindly processed by two authors Michael Schär and Lin Chen, respectively.

Reporting for specific materials, systems and methods

We require information from authors about some types of materials, experimental systems and methods used in many studies. Here, indicate whether each material, system or method listed is relevant to your study. If you are not sure if a list item applies to your research, read the appropriate section before selecting a response.

Materials & experimental systems

n/a	Involved in the study
☒	☐ Antibodies
☒	☐ Eukaryotic cell lines
☒	☐ Palaeontology
☒	☐ Animals and other organisms
☐	☒ Human research participants
☒	☐ Clinical data

Methods

n/a	Involved in the study
☒	☐ ChIP-seq
☒	☐ Flow cytometry
☒	☐ MRI-based neuroimaging

Human research participants

Policy information about studies involving human research participants

Population characteristics	All subjects are healthy volunteers. Age 27± 5; Gender: 2 male and 2 female subjects for the in-magnet plantar flexion exercise, while 5 male and 2 female subjects for the phosphate creatine optimization and high-resolution phosphate creatine mapping.
Recruitment	Participants were recruited through printed and electronic advertisements on notice boards in Johns Hopkins and Kennedy Krieger Institute.
Ethics oversight	The Johns Hopkins Institutional Review Board approved all human studies.

Note that full information on the approval of the study protocol must also be provided in the manuscript.
